# Integrative analysis of novel hypomethylation and gene expression signatures in glioblastomas

**DOI:** 10.18632/oncotarget.19171

**Published:** 2017-07-11

**Authors:** Anan Yin, Amandine Etcheverry, Yalong He, Marc Aubry, Jill Barnholtz-Sloan, Luhua Zhang, Xinggang Mao, Weijun Chen, Bolin Liu, Wei Zhang, Jean Mosser, Xiang Zhang

**Affiliations:** ^1^ Department of Neurosurgery, Xijing Institute of Clinical Neuroscience, Xijing Hospital, Fourth Military Medical University, Xi’an, Shaanxi Province, The People's Republic of China; ^2^ CNRS, UMR 6290, Institut de Génétique et Développement de Rennes (IGdR), Rennes, France; ^3^ Université Rennes1, UEB, UMS 3480 Biosit, Faculté de Médecine, Rennes, France; ^4^ CHU Rennes, Service de Génétique Moléculaire et Génomique, Rennes, France; ^5^ Plate-forme Génomique Santé Biosit, Université Rennes1, Rennes, France; ^6^ Case Comprehensive Cancer Center, Case Western Reserve University, Cleveland, Ohio, United States of America; ^7^ Department of Neurosurgery, No. 425 Hospital of the People's Liberation Army, San Ya, Hainan Province, The People's Republic of China; ^8^ Department of Neurosurgery, Arrowhead Regional Medical Center, Colton, California, United States of America

**Keywords:** glioblastomas, non-CpG island hypomethylation, gene network, molecular classification, precision oncology

## Abstract

Molecular and clinical heterogeneity critically hinders better treatment outcome for glioblastomas (GBMs); integrative analysis of genomic and epigenomic data may provide useful information for improving personalized medicine. By applying training-validation approach, we identified a novel hypomethylation signature comprising of three CpGs at non-CpG island (CGI) open sea regions for GBMs. The hypomethylation signature consistently predicted poor prognosis of GBMs in a series of discovery and validation datasets. It was demonstrated as an independent prognostic indicator, and showed interrelationships with known molecular marks such as *MGMT* promoter methylation status, and glioma CpG island methylator phenotype (G-CIMP) or *IDH1* mutations. Bioinformatic analysis found that the hypomethylation signature was closely associated with the transcriptional status of an *EGFR/VEGFA/ANXA1*-centered gene network. The integrative molecular analysis finally revealed that the gene network defined two distinct clinically relevant molecular subtypes reminiscent of different immature neuroglial lineages in GBMs. The novel hypomethylation signature and relevant gene network may provide new insights into prognostic classification, molecular characterization, and treatment development for GBMs.

## INTRODUCTION

Glioblastomas (GBMs) are the most frequent and devastating subtype of all gliomas and present as clinically and molecularly heterogeneous groups of diseases [1, 2]. Conventional prognostic classification based on histological features only provided limited clinical value as outcomes usually varied among patients with histologically similar tumors [3]. Like other cancers, GBMs are driven by a plethora of molecular alterations that may be characteristic of distinct biological phenotypes, clinical features, and treatment responses [4–7]. Comprehensive molecular profiling of clinically well-described GBM cohorts may provide additional information for improving patient management [8].

Genome-wide epignetic studies have showed that cancers are commonly featured by global hypomethylation of gene-poor DNA repeats and large hypomethylated blocks of gene regions concurrent with relevant CpG island (CGI) hypermethylation [9–11]. Those epigenetic aberrations may have crucial roles in tumor genesis and progression via the regulation of gene expression and chromatin structure [9]. Early efforts with candidate-gene approach have been directed towards searching powerful DNA methylation biomarkers with a focus on promoter-specific CGI hypermethylation [9, 12]. In GBMs, promoter hypermethylation in key genes (e.g., *MGMT*, *TIMP3*, *RASSF1A* and *p16INK4a*) have been identified as potential biomarker candidates, which may be helpful for improving risk classification or guiding treatment choice [13]. More recently, with the increasing popularity of high-throughput molecular detection technology [14], comprehensive assessment on GBM epigenome may greatly expand our knowledge on current epigenetic biomarker discovery, and be useful for improving personalized medicine.

In this study, by anlyzing high-throughput DNA methylation micorarray data, we report a novel prognostic signature based on DNA hypomethylation of three CpGs at non-CGI open sea regions for GBMs. Bioinformatic analysis of gene expression data shows that the hypomethylation signature is closely associated with the transcriptional status of an *EGFR*/*VEGFA*/*ANXA1*-centered gene network, which may biologically explain the observed survival difference of each risk subgroup, and provide new insights into the molecular and clinical understanding of GBMs.

## RESULTS

### Discovery and validation of a DNA hypomethylation signature for poor prognosis of GBMs

**Figure 1:**
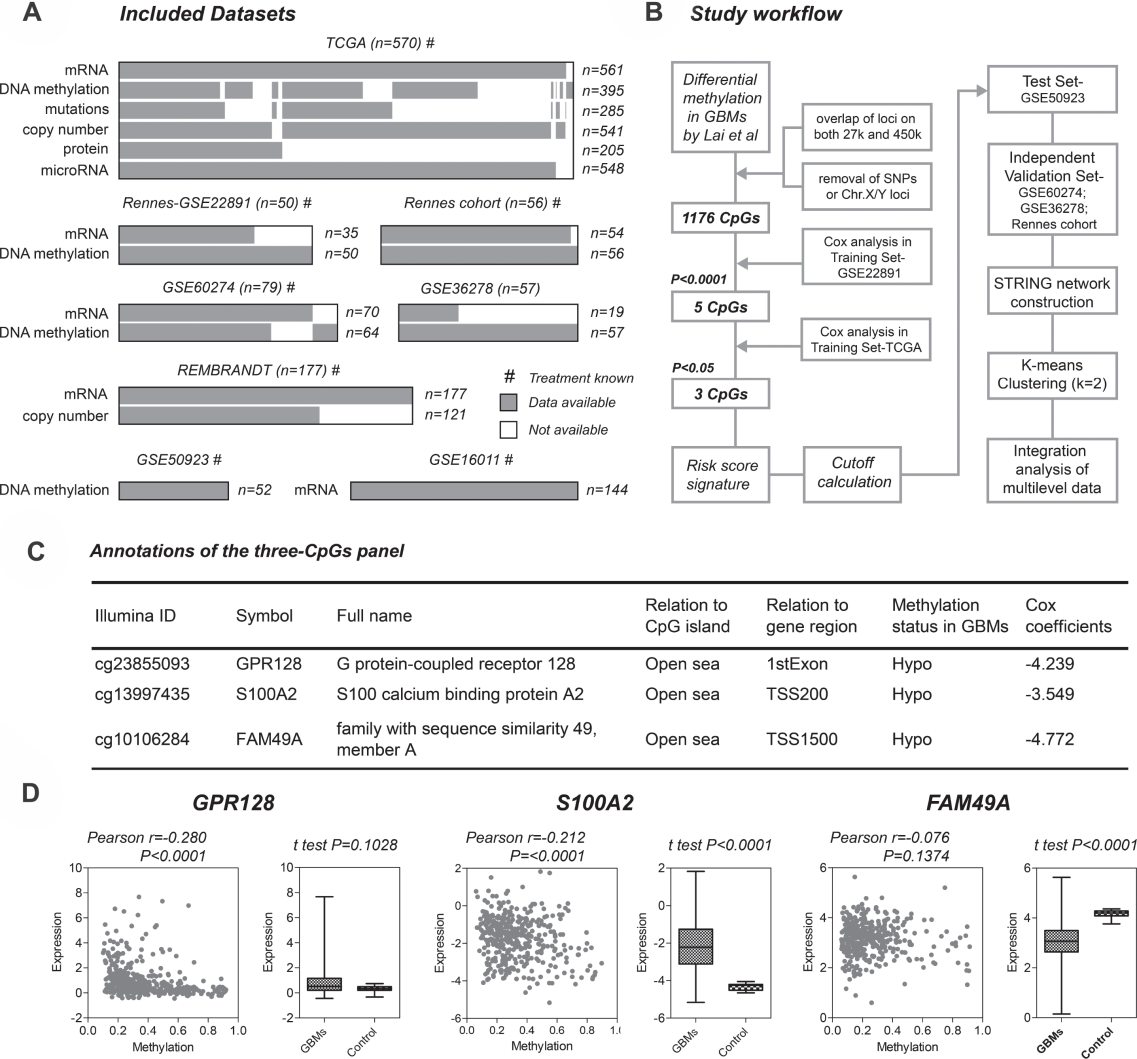
Identification of the novel three-CpGs signature for glioblastomas (GBMs) (**A**) all patient cohorts and molecular data sets that were included for the study. (**B**) schematic diagram of the entire workflow for the study. (**C**) characteristics of the three-CpGs panel; Cox coefficients were calculated within the training set – GSE22891. (**D**) the effects of DNA methylation on locus-specific gene expression across tumors (*left*) and expression levels between GBMs and non-tumor brain tissues (*right*) for each CpGs; molecular data of GBMs (n=386) and controls (*n* = 10) were obtained from TCGA.

The included datasets and study workflow are schematically shown in Figure 1A–1B, and patient characteristics are summarized in Supplementary Table 1. By employing a multi-step selection criterion, we identify a novel hypomethylation signature for GBM prognostication, which comprises of three CpGs interrogated on both Infinium 27 k and 450 k platforms: cg23855093 (*GPR128*), cg13997435 (*S100A2*) and cg10106284 (*FAM49A*) (Figure 1C). Interestingly, the CpGs are all located at non-CGI open sea regions (≥ 4000 bp far from relevant CGIs), and are all hypomethylated in GBMs. Losses of DNA methylation at those CpGs are all correlated with poorer prognosis as their Cox coefficients are all negative (Figure 1C). Regarding CpGs-specific gene expression, *S100A2* and *FAM49A* are differentially expressed in GBMs, and the expression levels of *S100A2* and *GPR128* are moderately correlated with open sea DNA methylation (Pearson r coefficients range from -0.3 to –0.2; *P* < 0.0001; Figure 1D).

**Figure 2:**
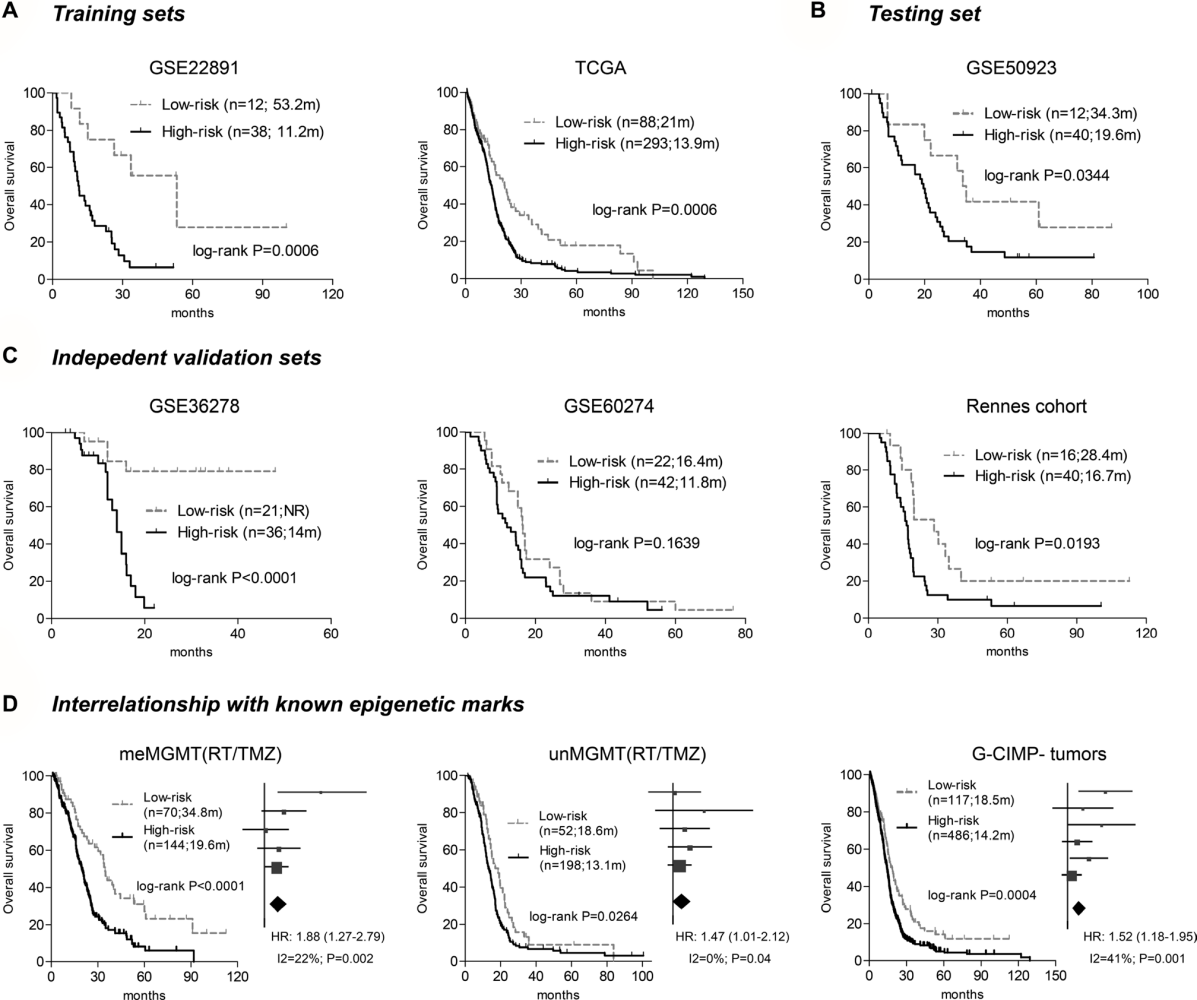
The survival correlation of the three-CpGs signature in each dataset (**A**) the hypomethylation signature predicted overall survival (OS) in two training sets – GSE22891 and TCGA. (**B**) It was also correlated with different OS in the testing test – GSE50923. (**C**) The three-CpGs signature was further validated in three independent validation cohorts, by yielding apparent OS difference in GSE36278 and Rennes cohort, and a trend for significance in GSE60274. (**D**) The three-CpGs signature was also able to identify patients with different prognoses within *MGMT* methylated tumors (left), unmethylated tumors (middle), and G-CIMP-negative tumors (right) among all available patients; the prognostic abilities were also confirmed by dataset-level meta-analysis, which was shown aside in a manner of forest plot.

Based on the three CpGs, a risk-score formula that is the sum of DNA methylation levels of each CpGs weighted by its Cox regression coefficients is constructed as follows: risk score = (–4.235 × β value of cg23855093 + (–4.765 × β value of cg10106284) + (–3.542 × β value of cg13997435). Using the cutoff calculated by *maxstat* R package (–5.047), the hypomethylation signature could assign each patient to a low-risk (with lower scores) or a high-risk (with higher scores) group from both training sets, TCGA [7] and GSE22891 [15], where low-risk patients are associated with significantly longer OS than high-risk ones (Figure 2A). The hypomethylation signature is further validated in the testing set (GSE50923 [16]), and in three independent validation set (GSE36278 [17], GSE60274 [18, 19], and Rennes cohort) by yielding apparent difference in OS between the assigned risk subgroups (Figure 2B–2C).

### The hypomethylation signature is an independent prognostic factor and has interrelationships with known molecular biomarkers

Univariate Cox regression analysis with all Rennes patients (GSE22891 [15] and Rennes cohort collectively) shows that age, glioma-CpGs island methylator phenotype (G-CIMP) status, TCGA gene expression subtypes, *MGMT* methylation status, and the hypomethylation signature are all significantly correlated with OS (Table 1). Multivariate Cox model further demonstrates the hypomethylation signature as an independent prognosticator (Table 1). Cox regression analysis yields similar results by using all available patients, which also indicates the treatment-independent prognostic nature of the hypomethylation signature (Supplementary Table 2).

**Table 1:** Results of Cox regression analyses in rennes cohorts

Variables	Univariate Cox model			Multivariate Cox model		
	HR	95%CI	*P* value	HR	95%CI	*P* value
Rennes cohorts (*n* = 106)^a^						
Patient age	1.026	1.005–1.049	0.016	1.031	1.007–1.056	0.011
KPS	1.000	0.986–1.013	0.942			
Three-CpGs signature	0.354	0.209–0.601	< 0.001	0.381	0.220–0.662	0.001
MGMT methylation status	2.567	1.650–3.994	< 0.001	2.835	1.756–4.576	< 0.001
G-CIMP status	4.901	0.681–35.261	0.114			
TCGA gene expression subtypes^b^	1.149	0.943–1.400	0.167			
Gender	0.866	0.558–1.343	0.519			
Extent of surgery (total/partial/biopsy)	0.642	0.437–0.944	0.024	0.549	0.358–0.841	0.006

HR = hazard ratio; CI = confidence interval; KPS = Karnofsky performance score; G-CIMP = gliomaCpG island methylator phenotype.

^a^Rennes cohorts included GSE22891 and Rennes cohort (*n* = 106).

^b^TCGA gene expression subtypes includes mesenchymal, classical, proneural, and neural subtypes.

In bold type were reported statistically significant results.

The hypomethylation signature also shows correlations with known molecular marks; the assigned low-risk groups had a higher frequency of *MGMT* methylated tumors (Fisher's exact test, *P* = 0.0004). Meta-analysis at individual-patient and dataset levels both shows that, among patients treated with radiation (RT) and temozolomide (TMZ), the hypomethylation signature has significant prognostic ability within subgroups of each *MGMT* status (Figure 2D). Moreover, we find that G-CIMP GBMs, a distinct epigenetic subgroup with favorable prognosis [6], are nearly exclusive in the low-risk groups, but the hypomethylation signature still has robust prognostic value in the subset of G-CIMP-negative tumors (Figure 2D). Finally, somatic mutations in *IDH1* (Fisher's exact test, *P* < 0.0001) and *ATRX* (Fisher's exact test, *P* = 0.0002) are significantly enriched in the low-risk tumors, while the unfavorable mesenchymal subtype appears to be evenly distributed among the risk tumors (Fisher's exact test, *P* = 0.5906). The hypomethylation signature also shows prognostic values among tumors with wild type *IDH1* or *ATRX*, as well as the subpopulations of mesenchymal and non-mesenchymal subtypes (Supplementary Figure 1).

### The hypomethylation signature is closely associated with the transcriptional status of an *EGFR/VEGFA/ANXA1*-centered gene network

**Figure 3:**
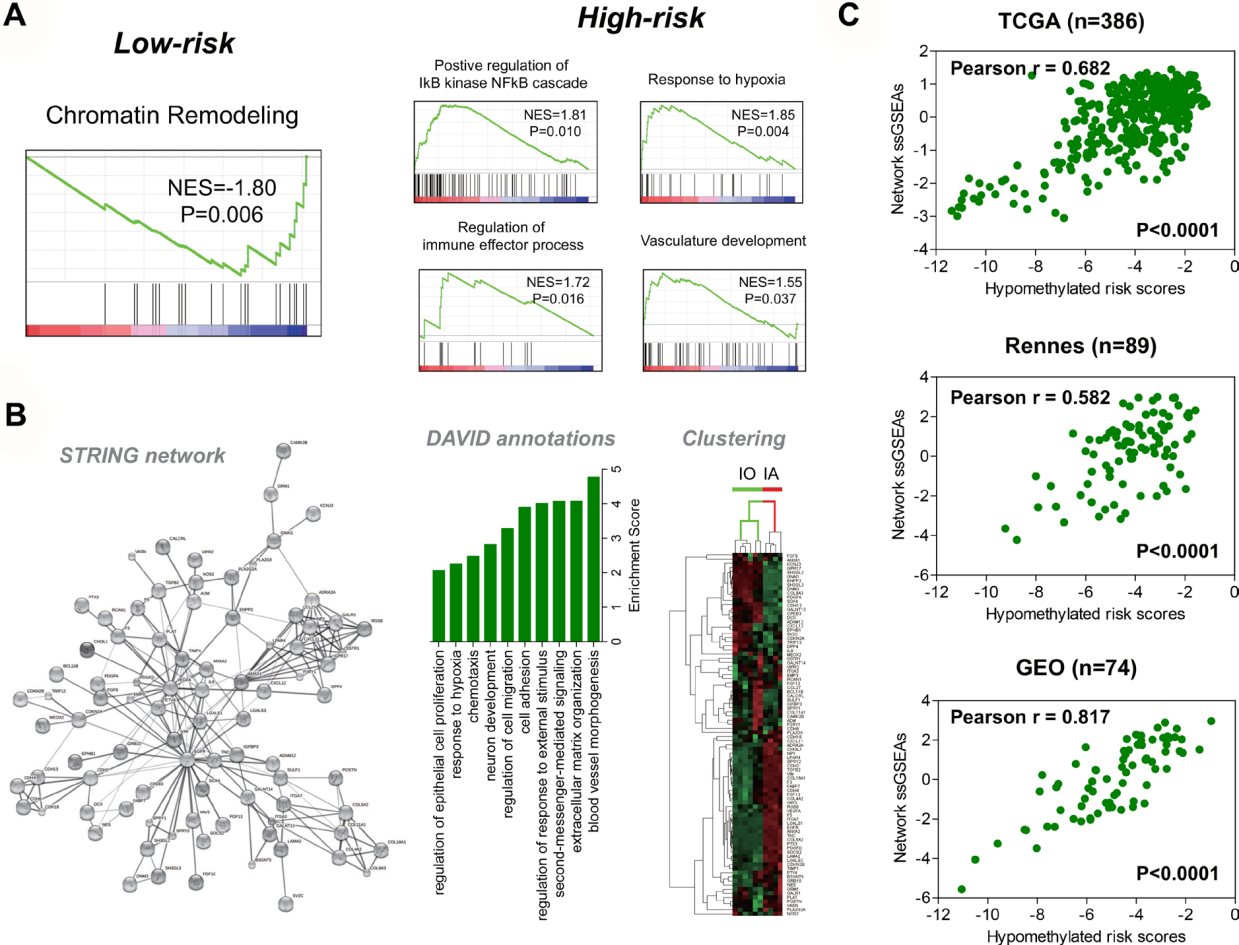
Functional relevance of the hypomethylation signature (**A**) GSEA enrichment plots for representative functional gene sets enriched in low-risk and high-risk tumors from TCGA. (**B**) based on the top differentially expressed genes (699 genes) between the risk groups in TCGA, a novel 88-gene interaction network was constructed by STRING database, which was centered on *EGFR* (25 connection nodes), *VEGFA* (16 connection nodes), and *ANXA1* (12 connection nodes; *left*); the top featured functional groups for the gene network classifiers were identified by DAVID database, showing that those genes were mostly involved in biological processes related to cancer and neural cell development (*middle*); each bar was indicated by the most representative annotations (with the smallest *P* value) for each functional groups, and was ordered by group enrichment score, that was the geometric mean of member's *p*-values in a corresponding annotation cluster; hierarchical clustering on the gene network classifiers clearly separated signatures of immature oligodendrocytes (IO) including non-myelinated oligodendrocytes and oligodendrocyte progenitor cells, from immature astroctyes (IA) (postnatal 1 to 8 days), also suggesting the relevance of the gene network to neural cell development (*right*). (**C**) Pearson correlation analysis showed that the risk scores of the hypomethylation signature were consistently and strongly in positive correlation with the expression scores of the gene network not only in the deriving TCGA, but also in two independent databases – Rennes (GSE22891 and Rennes cohort collectively) and GEO (GSE36278 and GSE60274 collectively); only samples with corresponding DNA methylation and gene expression data were analyzed.

Based on TCGA gene expression data, gene set enrichment analysis (GSEA) shows that high-risk tumors are enriched with many cancer-promoting gene sets relating to activation of NF-kB signaling, immune response, and pro-angiogenic signaling (Figure 3A and Supplementary Table 3). To explore the core network of transcriptional changes that may underlie the hypomethylation signature, we calculate the differentially expressed genes (DEGs) between the TCGA risk subgroups, and subject the top ten percent DEGs (699 genes, ranked by their log2 fold change values) to STRING database. The bioinformatic tool identifies an *EGFR*/V*EGFA*/*ANXA1*-centered gene interaction network, which comprises of an up-regulated gene set (55 genes) and a down-regulated gene set (33 genes) (Figure 3B and Supplementary Table 4). DAVID annotation analysis shows that the network genes mostly have roles in cancer-relevant (e.g., angiogenesis, extracellular matrix organization, and cell proliferation/migration) or neural cell developmental processes (e.g., neuron development; Figure 3B). Interestingly, we find that hierarchical clustering based on the gene network expression data could clearly distinguish murine immature astrocytes (IAs) from immature oligodendrocytes (IOs), which also suggest its relevance to neuroglial developmental processes (Figure 3B).

To confirm the correlations between the hypomethylation signature and the gene network, Pearson correlation analysis of two-level molecular data is performed on samples from Rennes, TCGA and Gene expression omnibus (GEO), which reveals the strong and positive correlation between the expression scores of the gene network (defined by single-sample GSEA; see in Materials and Methods) and the risk scores of the hypomethylation signature (Figure 3C). Similarly, GSEA shows the differential enrichment status of the down-regulated and up-regulated gene sets within each risk subgroups across each dataset (Supplementary Table 5).

### The *EGFR/VEGFA/ANXA1*-centered gene network defines two distinct clinically relevant molecular subclusters of GBMs

**Figure 4:**
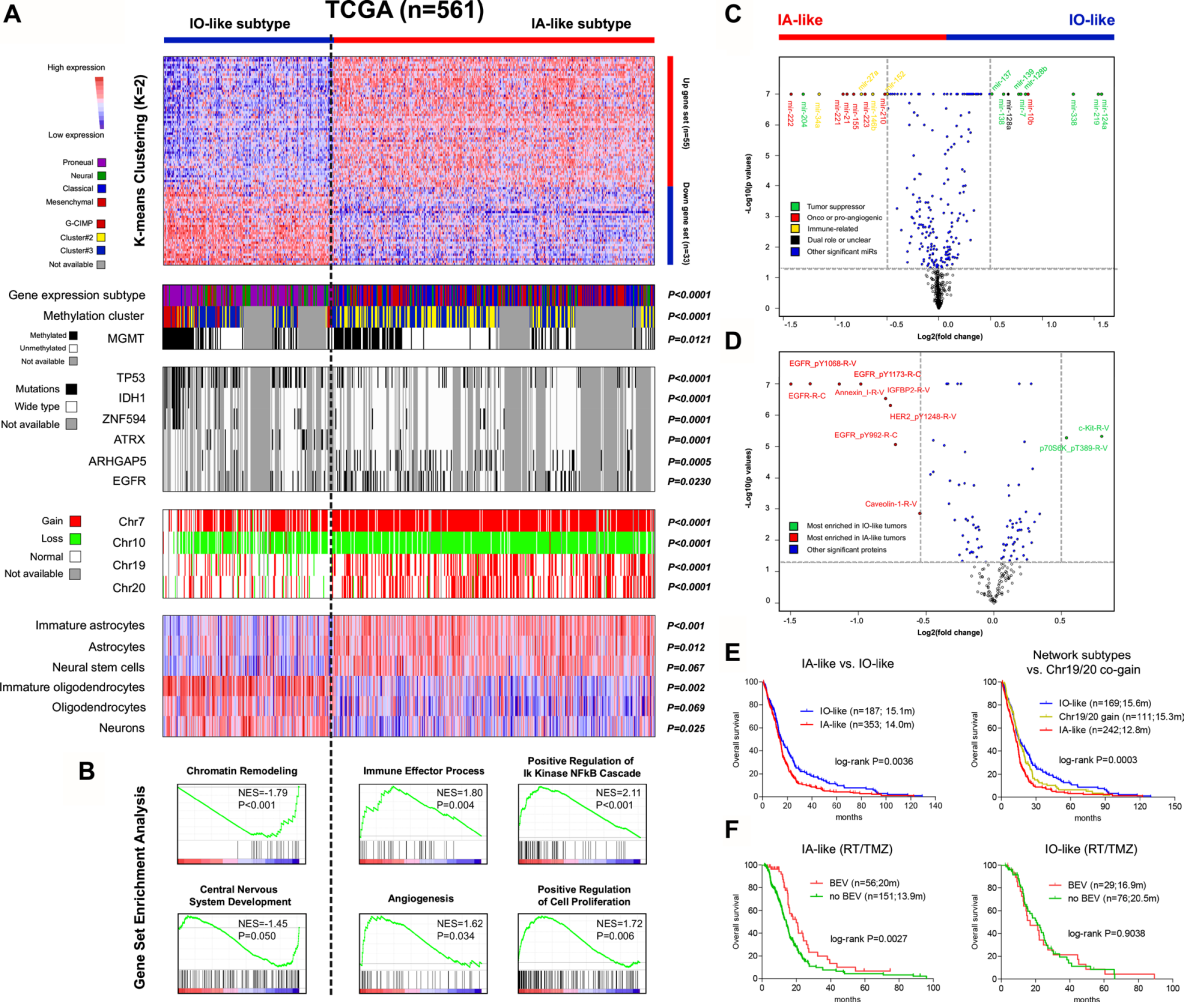
Molecular and clinical characterization of the two distinct subtypes of GBMs defined by the *EGFR/VEGFA/ANXA1*-centerred gene network using TCGA multi-dimensional data (**A**) the heat maps of K-means (k = 2) clustering on the gene network signature; each row represented a gene which was ordered according to the log2 fold change value calculated from TCGA; each column represented a sample; for each sample (*n* = 561), subgroup correlation, multi-level molecular features, and enrichment levels for signatures specific to distinct neural cell lineages were indicated; *P* values for Fisher’ exact test, Chi-square test, and GSEA were accordingly shown; (**B**) representative functional gene sets enriched in each subtype were also shown in a manner of enrichment plot; (**C**–**D**) the volcano plots of the differentially expressed microRNAs and proteins between the subtypes; the top-ranked ones (absolute log2 fold change > 0.5) were indicated; (**E**) the subtypes showed strong clinical correlations: the IO-like tumors were significantly associated with longer overall survival (OS) than the IA-like ones (*left*); incorporation of concurrent gain of chr.19/20 further identified a subgroup with more unfavorable prognosis within the IA-like tumors (*right*); (**F**) among TCGA patients treated with radiation (RT) and temozolomide (TMZ), the use of bevacizumab (either first-line or at progression) did confer a clear OS benefit to those with the IA-like tumors but was associated with similar OS among the IO-like tumors; recurrent, secondary or previously treated cases were excluded in this interaction analysis.

Consensus k-mean clustering on the gene network signature clearly assigns TCGA tumors into two main clusters with clustering stability decreasing for k = 2 to 6 (Figure 4A and Supplementary Figure 2). The boundaries of the two clusters are statistically significant (*P* < 0.0001). Given the potential relevance of the gene network to neural cell development, we perform GSEA to evaluate the enrichment of signatures characteristic of different neuroglial lineages [20, 21] within the clusters. GSEA shows that the two clusters are mostly featured by IOs and IAs signatures respectively (Supplementary Table 6). In addition, the clusters show moderate enrichments in signatures of other neural cell types: the cluster with IA signature is also enriched in signatures of mature astrocytes and neural stem cells whilst the cluster with IO signature is enriched in signatures of mature oligodendrocytes and neurons (Figure 4A and Supplementary Table 6). The clusters and relevant features are also observed in non-TCGA samples (Supplementary Figure 2 and Supplementary Table 6). The overlap of some gene classifiers and the correlation to specific neural cell types may result in the similarity of the gene network clusters to the gene expression subtypes by Verhaak et al. [4]: the proneural and neural subtypes and the classical and mesenchymal subtypes were respectively enriched in each cluster (Figure 4A). However the gene network signature could identify tumors with different states of immature neural cell signatures within each Verhaak subtype (Supplementary Figure 3). We therefore name the clusters in accordance with their neuroglial genesis activity respectively: the IO-like and the IA-like subtypes.

To characterize the two clusters, a comprehensive integrative analysis of clinical and molecular data from TCGA is performed:

### Transcriptional level

GSEA shows the differential functional profiles between the clusters: the IA-like cluster is enriched in cancer-promoting signatures relating to immune response, NF-kB activation, pro-angiogenic signaling and proliferative pathways, whilst the IO-like cluster is enriched in signatures of chromatin remodeling and normal brain development (Figure 4B and Supplementary Table 7). The functional features are also observed in non-TCGA samples (Supplementary Figure 4 and Supplementary Table 7).

### Genetic level

MutSigCV analysis shows the differential mutational profiles between the clusters: 301 and 473 genes respectively are significantly mutated in the IO-like and IA-like clusters, and 25% and 52% are cluster-specific (Supplementary Table 8). Among them, mutations in *EGFR* and *ARHGAP5* are significantly enriched within the IA-like cluster (Fisher’ exact test, *P* < 0.05) whilst mutations in 53 genes including *IDH1*, *ATRX*, *TP53* and *ZNF594* are enriched within the IO-like cluster (Fisher’ exact test, *P* ≤ 0.0001; Figure 4A). The gene network clusters also exhibit distinct chromosomal alterations: gain of Chr7 and loss of Chr10 are more frequently seen in the IA-like cluster, whilst loss of Chr11, Chr13q, Chr14q and Chr15q are more frequently seen in the IO-like cluster (Figure 4A and Supplementary Figure 5). Moreover, gain of Chr19 and Chr20 are found to be nearly exclusive in the IA-like cluster (Figure 4A). The cluster-specific chromosomal features are also seen in REMBRANDT samples (Supplementary Figures 4–5).

### Epigenetic level

Correlation with DNA methylation clusters by Noushmehr et al. [6] shows that the IO-like and IA-like clusters are respectively enriched with G-CIMP and cluster#2 GBMs whilst cluster#3 tumors are evenly distributed across the clusters (Figure 4A). The IO-like cluster has a higher frequency of methylated *MGMT* promoter (Fisher's exact test, *P* = 0.0121; Figure 4A). Using TCGA miRNA Microarray data, we identify 218 differentially expressed microRNA (DEmiRs; 110 up- and 108 down-regulated) between the clusters (Supplementary Table 9). The IA-like tumors are featured by up-regulation of oncogenic and pro-angiogenic microRNAs (e.g., mir-222/221, mir-21, and mir-155) [22–24] and immune-modulating microRNAs (e.g., mir-34a, mir-27a, and mir-146b) [25–27] whilst the IO-like tumors are enriched with tumor suppressor microRNAs (e.g., mir-124a, mir-219, and mir-338), some of which also have roles in neural cell differentiation [28–31] (Figure 4C).

### Protein level

The gene network clusters also exhibit distinct downstream protein alterations; 43 and 43 proteins were up- and down- regulated in the IA-like vs. IO-like tumors (Figure 4D, and Supplementary Table 10). The IO-like tumors show elevation of a pro-oncogene (c-Kit) and activation of known signaling pathways such as MAPK (e.g., B-Raf, K-Ras, MEK1, JNK, Tau, and Stathmin 1), PI3K/Akt/mTOR (e.g., Tuberin, GSK-3β, p70S6K, 4EBP1, and p27), and P53 (e.g., cyclin B1, cyclin D1, cyclin E1, p53, cleaved-caspase-9, and Chk2). Regarding the IA-like tumors, phospho- and/or total protein levels of *EGFR* and *ANXA1* are increased as anticipated, as well as focal adhesion (e.g., IGF-1R-β, Caveolin-1, HER2, Collagen VI, FAK, and fibronection) and VEGF signaling pathway (e.g., VEGFR2, MAPK, paxillin, Src, p38, and Cox-2).

### Clinical level

Besides the distinct molecular features, the gene network clusters show strong clinical correlations. In TCGA, the IA-like tumors are significantly associated with shorter OS than the IO-like ones (Figure 4E). Multivariate Cox model shows the gene network clusters as an independent prognosticator in the context of age, G-CIMP status, *MGMT* methylation status, and treatments (Supplementary Table 11). Interestingly, the concurrent gain of Chr19/20, a newly identified favorable genetic marker for non-G-CIMP tumors [5], is found to be nearly exclusive in the unfavorable IA-like cluster (104 of 114; Figure 4A). The incorporation of the genetic marker to the gene network clusters further identifies a more unfavorable subgroup of IA-like tumors without co-gain of Chr19/20 (Figure 4E). The clinical features are also seen in non-TCGA datasets (Supplementary Figure 4).

Of note, the integrative analysis reveals apparent and concordant activation of pro-angiogenic signaling at multiple molecular levels in the IA-like cluster. Therefore it is reasonable that the clusters may have differential responses to anti-angiogentic therapy. Using the TCGA drug data, we find that, among patients with RT/TMZ, the addition of bevacizumab (either first-line or at recurrence; a humanized monoclonal antibody against *VEGFA* [32, 33]) do confer a clear OS benefit to patients within the IA-like cluster, but is associated with similar OS within the IO-like cluster (Figure 4F). Of note, the results are not conclusive, and should be validated within prospective trials.

## DISCUSSION

Epigenetic marks and DNA methylation in particular have been the leading candidates for biomarker discovery as they have advantages over genetic- or expression-based information: reliable DNA samples, stable altering patterns, multilevel biological information, and drug-induced reversibility [34]. There have been precedents of DNA methylation marks and especially those at promoter-specific CGI regions as more powerful parameters than other molecular information for cancer diagnosis and prognosis, among which *MGMT* hypermethylation for better outcome to TMZ is the most remarkable one [12]. The clinical value of DNA methylation aberrations outside the CGI-relevant gene regions however has been largely overlooked due to the long-standing focus on cancer-linked CGI hypermethylation [9]. In this study, we report the first epigenetic biomarker of non-CGI open sea hypomethylation for GBM prognostication. The risk-score based epigenetic classifier shows robust prognostic value as it has been validated in different GBM cohorts. Moreover, the hypomethylation signature shows good interrelationships with the current widely-used biomarkers such as G-CIMP status (or *IDH1* mutations) and *MGMT* methylation status. Therefore, the incorporation of the hypomethylation signature into current molecular classification could be logically practical and be helpful for optimizing risk stratification of GBM patients. Although it is of promising value, there still have technical issues to be solved before its application in clinical routine testing. The risk-score signature is developed based on DNA methylation microarray data, which however could not be easily available for routine practice. Quantitative pyrosequencing is a well-established and widely used method for DNA methylation detection [35]. Comparative study has showed very good congruence of DNA methylation data from Illumina DNA methylation array with pyrosequencing data [35]. The conventional technique may thus represent an alternative for the three-CpGs methylation profiling in clinical setting. Independent validation study will be required for adjusting the microarray-based signature to a pyrosequencing-based one because that inconsistence of DNA methylation data for individual loci between the two methods really exists.

By far limited data have been explored for biological and clinical impacts of gene hypomethylation in cancers. Early studies focusing on cancer-linked hypomethylation at gene-poor DNA repeats suggested that those DNA methylation aberrations may lead to instability of chromatin structure, reactivation of transposable elements, and *in trans* modification of relevant gene expressions [36]. Within gene regions, about 40% of all human genes have no CGIs at their promoters [37]. Recent genome-wide epigenetic studies of common solid tumors [11, 38] showed that tumor-specific hypomethylated loci favor non-CGI promoters and open sea regions in particular, and are usually associated with large-scale hypomethylated blocks [11]. Like CGI hypermethylation, DNA hypomethylation may contribute to tumors via affecting individual gene expression (e.g., re-expression of pro-oncogenic genes) [36]. However, studies have showed that DNA methylation has overall limited control for non-CGI promoters, and the expression levels of hypomethylated genes are much variable, as a result of the spatial and temporal balance of various transcription regulatory activators and repressors [11, 37] . For blocks of multiple hypomethylated loci, they may have a broader effect by affecting heterochromatin structures in normal cells, and making them become euchromatic in cancer cells, as well as leading to loss of epigenetic regulation, and resulting in hyper-variability of gene expression [38]. In our study, we find that the three open sea CpGs showed limited impact on individual gene expression, which may be unlikely to fully explain the robust survival difference conferred by the hypomethylation signature. Actually, the three open sea CpGs are all located within the large hypomethylated blocks reported by Timp et al. [11]. It is reasonable to assume that the three-CpGs panel may be part of GBM-specific large hypomethylated blocks, which may have wide influences on tumor transcriptome, and its clinical and biological impacts may be associated with the expression of sets of functionally interacted genes, not limited to locus-specific gene expression. Following the assumption, we identify an 88-gene core network centered by *EGFR*, *VEGFA* and *ANXA1* that are closely associated with the hypomethylation signature. Most of the network genes have been shown to have roles in key biological processes of glioma and neural cell development (Supplementary Table 4). Clustering based on the gene network expression data could stratify GBMs into two distinct clusters of tumors with similar gene network expression patterns. Integrative analysis demonstrates the distinct molecular and clinical features of the defined clusters at multi-platform levels. The reproducibility of the clusters and relevant features across each dataset suggests that it is unlikely that the new clusters are a spurious finding due to technical artifact or sample bias. All the data support that the gene network may have important roles in GBM biology and prognosis. Therefore, it is reasonable to believe that the core gene network may be key molecular effectors that could biologically explain the survival impact of the hypomethylation signature in GBMs. However, it should be noted that our study only demonstrates the close correlation between the two-level molecular signatures, the causality, or their exact molecular interaction, remains further investigation.

The characterization of the gene network clusters provides novel insights into the understanding of molecular heterogeneity of GBMs. Rather than specifically correlating to neuroglial lineages at mature stage, which has been shown in the Verhaak subtypes [4], the expression patterns of the gene network could identify subsets of GBMs reminiscent of distinct immature neural cell types. The clusters may be more consilient with the hypothesis that GBMs are likely to be derived from neural progenitor cells. Moreover, instead of a purely data-driven approach, the gene network is constructed by a bioinformatics-based approach, which takes advantages of validated molecular data from STRING database [39]. The gene network is actually a collection of functionally relevant genes that may collaboratively have roles in determining tumor phenotypes. The expression patterns of the whole gene network can serve as a more reliable indicator than individual gene expressions to predict such phenotype. In our study, we find that the two clusters exhibit stable but distinct functional features across different datasets: the IA-like tumors appear to be more aggressive and less differentiated as they are featured by activation of NF-kB signaling, immune response, and pro-angiogenic signaling, and down-regulation of neural developmental signatures. It is believed that those cluster-specific features may serve as potential therapeutic targets for differential treatment towards GBMs, among which the abnormal activation of pro-angiogenic pathways represents the most promising one. Within TCGA samples, we observe differential treatment outcomes of bevacizumab-contained therapy within the gene network clusters, supporting its implication in guiding anti-angiogenic therapy for GBMs. The finding is encouraging especially after the disclosure of two Phase III trials failing to justify the survival benefits of bevacizumab for unselected primary GBM patients [32, 33]. Of note the result should be conservatively interpreted due to apparent study limitations (e.g., incomplete drug data, second-line bevacizumab in most cases, and retrospective design). Prospective validation in randomized trials of first-line bevacizumab is needed for final conclusion.

Accumulating studies including ours have highlighted the high relevance of gene hypomethylation to cancer biology and prognosis [36]. Those findings may raise new concerns against the current demethylation agents for anti-cancer therapy as they may have adverse therapeutic effects by exacerbating cancer-linked gene hypomethylation. Therefore novel epigenetic modifiers that have reversed effects on DNA hypomethylation may be promising therapeutic agents in combination with current demethylation drugs.

In summary, our study takes advantage of the current high-throughput DNA methylation detection platform, and provides initial data on molecular and clinical relevance of gene hypomethylation in GBMs.

## MATERIALS AND METHODS

### Rennes cohorts

Totally fifty six adult patients (aged ≥ 18 years old) with newly diagnosed GBMs were collected from the Neurosurgery Departments of Rennes and Angers University Hospitals between 2004 and 2010 (Rennes cohort). All patients were homogenously treated with Stupp regimen [40]. Snap-frozen samples were collected, following informed consent, in accordance with the French regulations and the Helsinki Declaration. DNA was extracted using the NucleoSpin TissueKit (Macherey Nagel). The quality of DNA samples was assessed by electrophoresis in a 1% agarose gel. DNA methylation profiling was done with Infinium HumanMethylation450 k platform (Illumina Inc.). The novel *BMIQ* (Beta MIxtureQuantile dilation) algorithm was used for intra-array adjustment [41]. Methylation level of each interrogated CpGs locus is summarized as β value which provides a continuous and quantitative measurement of DNA methylation, ranging from 0 (completely unmethylated) to 1 (completely methylated). Gene expression profiling was done with the Agilent Whole HumanGenome 8 × 60 K Microarray Kit (Agilent Technologies). Microarray data were log_2_ transformed, and normalized (scale 50th percentile and baseline transformation) within GeneSpring GX software (Agilent Technologies). The genomic region spanning wild-type R132 of IDH1was analyzed by direct sequencing as previously described [15]. A published cohort of fifty newly diagnosed GBMs (Stupp regimen) from the Neurosurgery Departments of Rennes and Angers University Hospitals was also included with available genome-wide DNA methylation (GSE22891; Infinium27k platform, Illumina Inc.) and gene expression data (GSE22891; Agilent 4 × 44 K Microarray Kit, Agilent Technologies) [15].

### Public datasets

### The cancer genome atlas (TCGA)

Multi-platform molecular and clinical data from 570 GBM samples were downloaded from TCGA data portal [7]. Multi-level data sets included 1) level 3 customized AgilentG4502A_07 Microarray data for gene expression; 2) level 3 Infinium 27 k or 450 k Array data for DNA methylation; 3) level 2 Whole Exome Sequencing data for somatic mutation; 4) level 3 Affymetrix Genome-wide Human SNP6.0 Array data for copy number; 5) level 3 Agilent 8×15K Human miRNA Microarray data for microRNA, and 6) level 3 Reverse Phase Protein Array data for protein targets. In addition, gene expression data from ten non-tumor brain samples were obtained as controls [7].

### Repository of molecular brain neoplasia data (REMBRANDT)

A total of 177 GBM samples were retrieved from REMBRANDT [42]. Two-level data sets included 1) Affymetrix Human Genome Plus2.0 Microarray data for gene expression; and 2) Affymetrix100K SNP Array data for copy number data.

### Gene expression omnibus (GEO)

Four datasets of GBMs were also obtained from GEO, including a Switzerland cohort (*n* = 79; GSE60274 for DNA methylation and GSE7696 for gene expression; recurrent samples were excluded; ref. [18, 19]), a Germany cohort (*n* = 57; GSE36278 for DNA methylation and GSE36245 for gene expression; tumors harboring mutations in *H3F3A* and those from TCGA were excluded; ref. [17]), a American cohort (*n* = 52) with only DNA methylation data (GSE50923; ref. [16]) and a European cohort (*n* = 144) with only gene expression data (GSE16011; ref. [3]).

Of note, among the heterogeneous datasets of gliomas of all grades and ages, only patients with age over 18 years old, and a histological diagnosis of GBMs, were included in this study. Patients with a follow-up data ≥ one month were included for survival analysis, to reduce the bias caused by non-cancer death.

### Across-dataset (or platform) microarray data processing

For DNA methylation data, batch effects caused by different datasets and different platforms were adjusted by a non-parametric empirical Bayes approach (*ber* package) [43]. For gene expression data, expression values represented by multiple probes (or probe sets) were collapsed by taking the mean value of the set of probes (or probe sets). Gene expression datasets were standardized independently by z-score transformation, in which expression values of each gene were transformed to have a mean of zero and a standard deviation (SD) of one [17]. Missing values were imputed by *impute* package within R software.

### Construction of a risk-score signature from reported differentially methylated CpGs in GBMs

The list of 1548 differentially methylated CpGs between GBMs and non-tumor brain tissues from both TCGA and GSE50923 (*1548 CpGs*) reported by Lai et al [16] was downloaded as GBM-specific CpGs candidates. After removal of those targeting the sex chromosomes, those containing a single-nucleotide polymorphism within five base pairs or the targeted loci, and those not interrogated on both the 27 k and 450 k platforms, 1176 CpGs were kept for subsequent analysis. The training-validation approach was employed to construct a prognostic signature. First, univariate Cox regression analysis with permutation correction was performed using methylation levels of the GBM-specific loci and overall survival (OS) from the first training set – GSE22891. The top significant prognostic loci (5 CpGs; *P* < 0.0001) were then subjected to Cox regression model within the second training set – TCGA. Three of the five loci remained significant in TCGA (*P* < 0.05), and were then combined using a risk-score model. Cox regression coefficients of each CpGs were calculated from the clinically homogeneous set - GSE22891. The optimal cutoff to stratify different risk tumors was determined by *maxstat* package within the both training sets [44]. Bioinformatic analysis

GSEA was run to evaluate functional profiles between defined subgroups on reported gene sets from Molecular Signature Database (MSigDB) [45], with nominal *P* value ≤ 0.05 for significance. DEGs were computed by two-sample t test with a parametric *P* value ≤ 0.05 being significant. STRING database [39] was run to construct gene interaction network from the top DEGs, with the input options “experiment”, “co-expression”, “database”, “textmining” and “high confidence (0.700)”. The correlation of the hypomethylated signature and gene network was assessed by Pearson correlation analysis, with the risk scores of the hypomethylated signature and the expression scores of the gene network as variables [4]. The expression scores were defined as the z-score transformed ssGSEA projections of the up-regulated gene set minus the z-score transformed ssGSEA projections of the down-regulated gene set in each dataset [4]. DAVID database was run to provide functional annotations for input gene list [46].

### Integrative analysis of multi-dimensional molecular data

Unsupervised clustering was performed on the E*GFR/VEGFA/ANXA*-centered gene network. Increasing values of k (2 to 6) were used to identify optimal segregation. Cluster significance was evaluated using *sigclust* package [47]. Multi-level data sets were analyzed by the following statistical models: 1) copy number data were analyzed by GISTIC2.0 [48], with amplitude threshold being ± 0.2, and others being default; 2) somatic mutations were analyzed by MutSigCV [49] with false discovery rate (FDR) *q*-value ≤ 0.05 for significance; and 3) differentially expressed microRNAs and proteins (DEPs) were computed by two-sample *t* test. Both GSEA and ssGSEA were performed to assess the enrichment of signatures characteristic of different neuroglial lineages: classifiers of neurons, (immature) astrocytes and (immature) oligodendrocytes were generated from a mouse central nervous system developmental dataset (GSE9566; ref. [20]); classifiers of neural stem cells were retrieved from a co-expression gene module in the adult human subventricular zone [21]. The TCGA gene expression subtypes were predicted by Binary tree classification prediction using the 840 classifiers reported by Verhaak et al [4]. G-CIMP and relevant methylation clusters were determined by k-means (k = 3) clustering on the 1503 probes reported by Noushmehr et al [6]. *MGMT* promoter methylation status was determined by a logistic regression model based on two CpGs, i.e., cg12434587 and cg12981137 [50].

### Statistical analysis

Pearson correlation analysis was performed to evaluate the correlation between DNA methylation and gene expression. Hierarchical clustering analysis was performed within GenePattern (http://software.broadinstitute.org/). The distributions of known molecular features with respect to each subgroup were tested by Fisher's exact or Chi-square test. OS were estimated by the Kaplan-Meier Method, and compared by log-rank test. Multivariate Cox regression analysis was used to evaluate the independence of potential prognosticators, only incorporating variables that are significant in univariate Cox model. Pooled analysis of hazard ratios (HR) at dataset level was done by the inverse-variance method. The application of either fixed- or random-effect models was based on the intra-dataset heterogeneity which was calculated by I-square statistic with I-square > 50% being significant. All the calculations were done within SPSS and R software and *P* values ≤ 0.05 for significance were used.

### Ethical

All procedures performed in studies involving human were in accordance with the ethical standards of the institutional research committee and with the 1964 Helsinki declaration and its later amendments or comparable ethical standards.

## SUPPLEMENTARY MATERIALS FIGURES AND TABLES














