# Supplementary material for: Integrative analysis of novel hypomethylation and gene expression signatures in glioblastomas
Source: Oncotarget. 2017 Jul 11;8(52):89607–19. doi: 10.18632/oncotarget.19171 (PMC5685695; doi:10.18632/oncotarget.19171)
Supplement: Supplementary file 1 [file oncotarget-08-89607-s001.pdf]

Integrative analysis of novel hypomethylation and gene expression signatures in glioblastomas

SUPPLEMENTARY MATERIALS

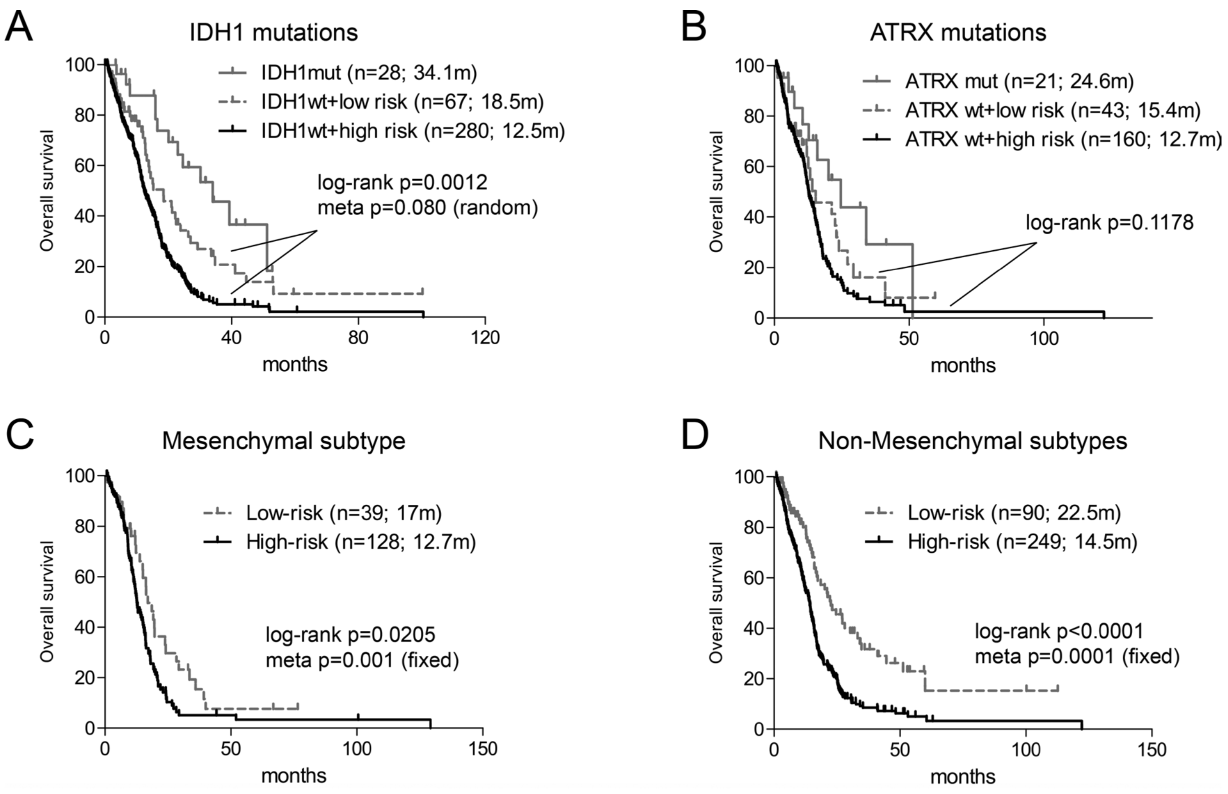

Supplementary Figure 1: The survival correlation of the three-CpGs signature with (**A**) *IDH1* mutations, (**B**) *ATRX* mutations, (**C**) Mesenchymal subtype and (**D**) Non-mesenchymal subtypes; the survival was compared by log-rank test at individual patient level and by meta-analysis at dataset-level; meta-analysis was not performed for *ATRX* mutations, which are only available for TCGA samples.

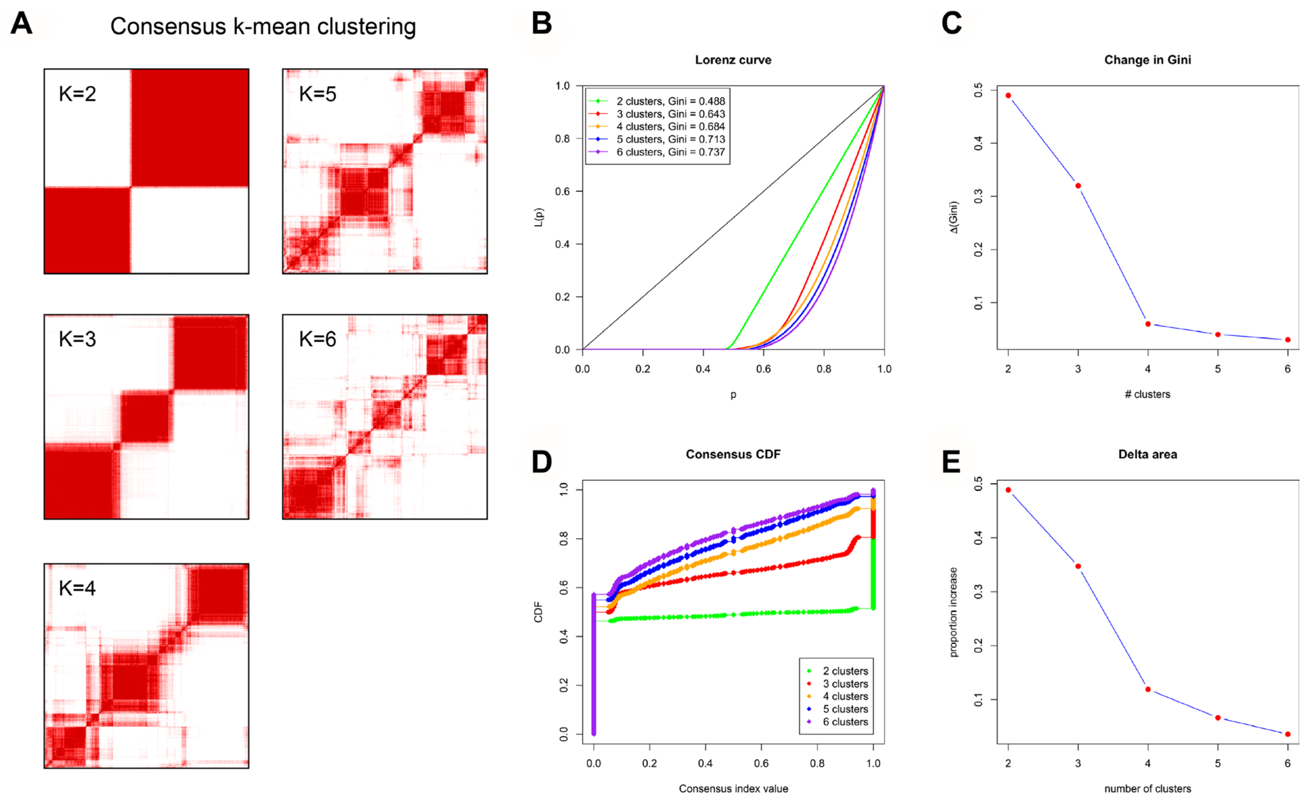

Supplementary Figure 2: The robustness of the gene network clusters in TCGA. (**A**) consensus clustering matrix of tumor samples for k = 2 to k = 6; (**B**) Lorenz curve for k = 2 to k = 6; (**C**) change value in Gini for k = 2 to k = 6; (**D**) consensus clustering CDF for k = 2 to k = 6; (**E**) relative change in area under CDF curve for k = 2 to k = 6.

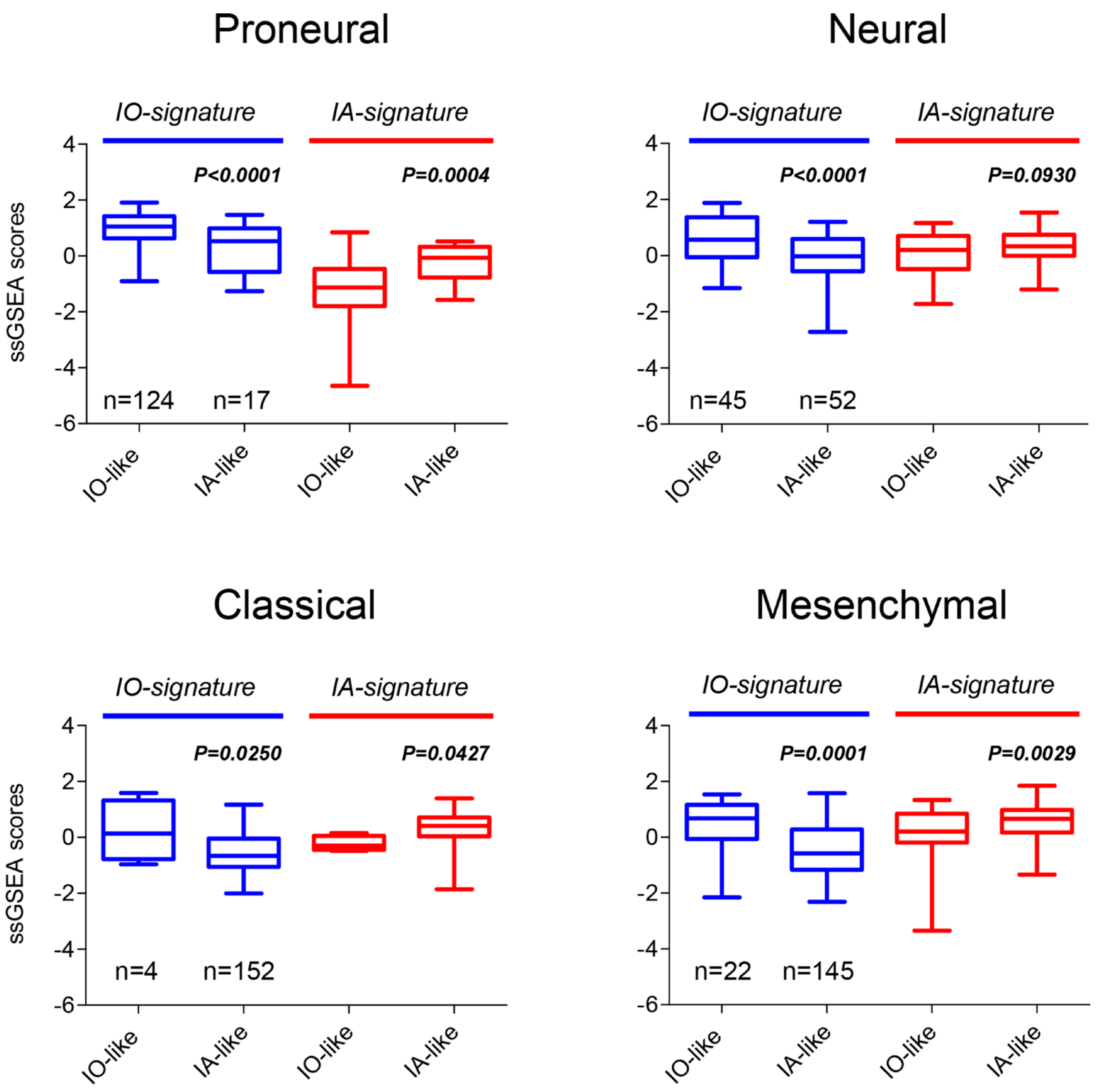

Supplementary Figure 3: Comparison of enrichment scores (ssGSEA) for signatures characteristic of immature oligodendrocytes (IOs) and immature astrocytes (IAs) between the IO-like and IA-like subtypes within each gene expression subtype by Verhaak et al. in TCGA.

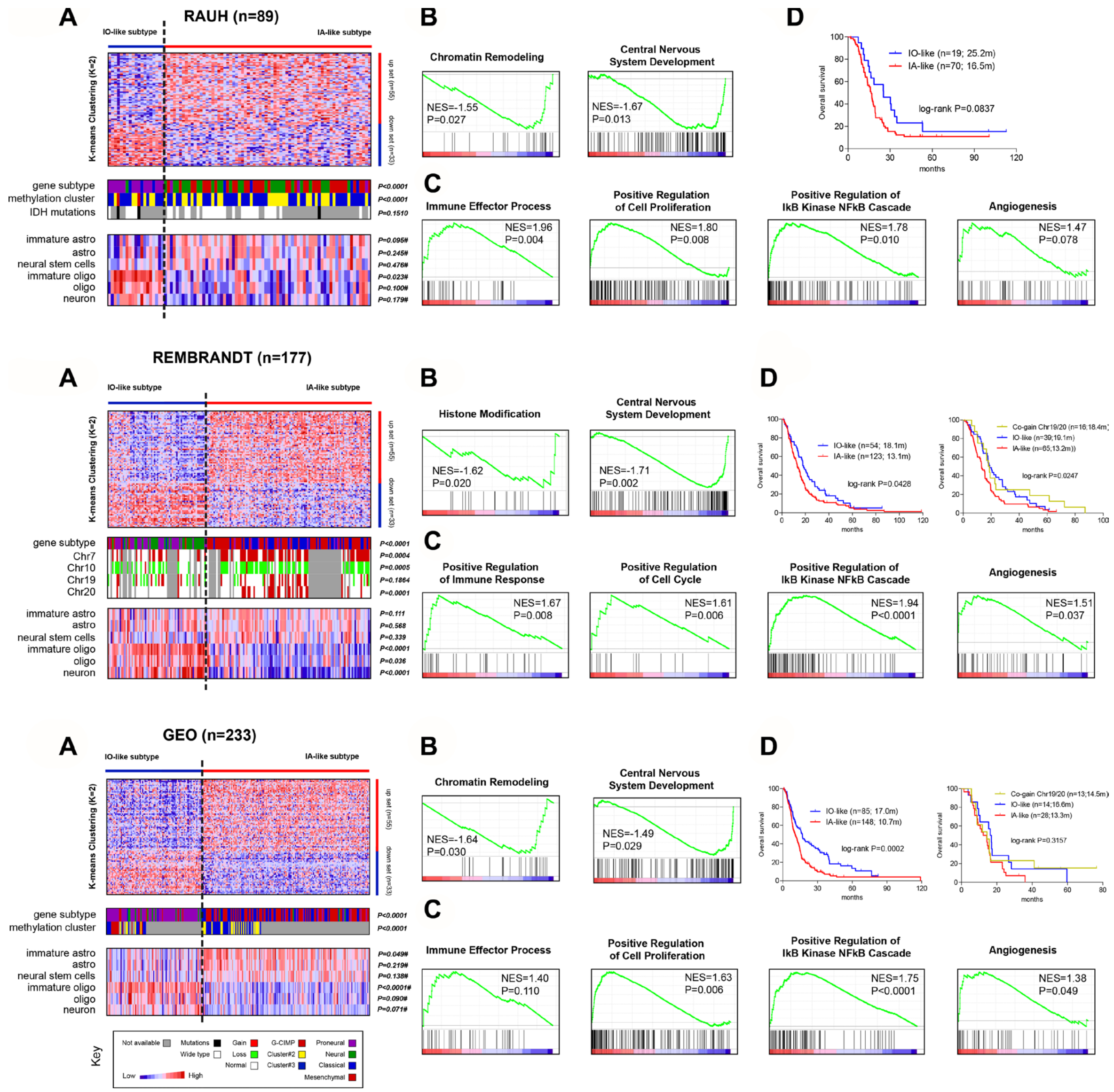

Supplementary Figure 4: Molecular and clinical characterization of the gene network subtypes within RAUH (GSE22891 and Rennes cohort collectively), REMBRANDT, and GEO (GSE16011, GSE7696, and GSE36245 collectively). (**A**) the heat maps of K-means (K = 2) clustering on the gene network signature; each row represents a gene; and each column represents a sample; available molecular subgroups, multi-dimensional molecular features, and single-sample enrichment scores for signatures of neural cell lineages were indicated for each sample; P values for Fisher' exact test, Chi-square test and GSEA were indicted; # indicated P values of GSEA for Rennes cohort in RAUH, and for GSE16011 in GEO; (**B**) the representative GSEA enrichment plots for the IO-like subtypes; (**C**) the representative GSEA enrichment plots for the IA-like subtypes; (**D**) the survival relevance of the subtypes or in combination with the concurrent gain status of chr.19/20; the genetic mark was available for REMBRANDT and GSE7696.

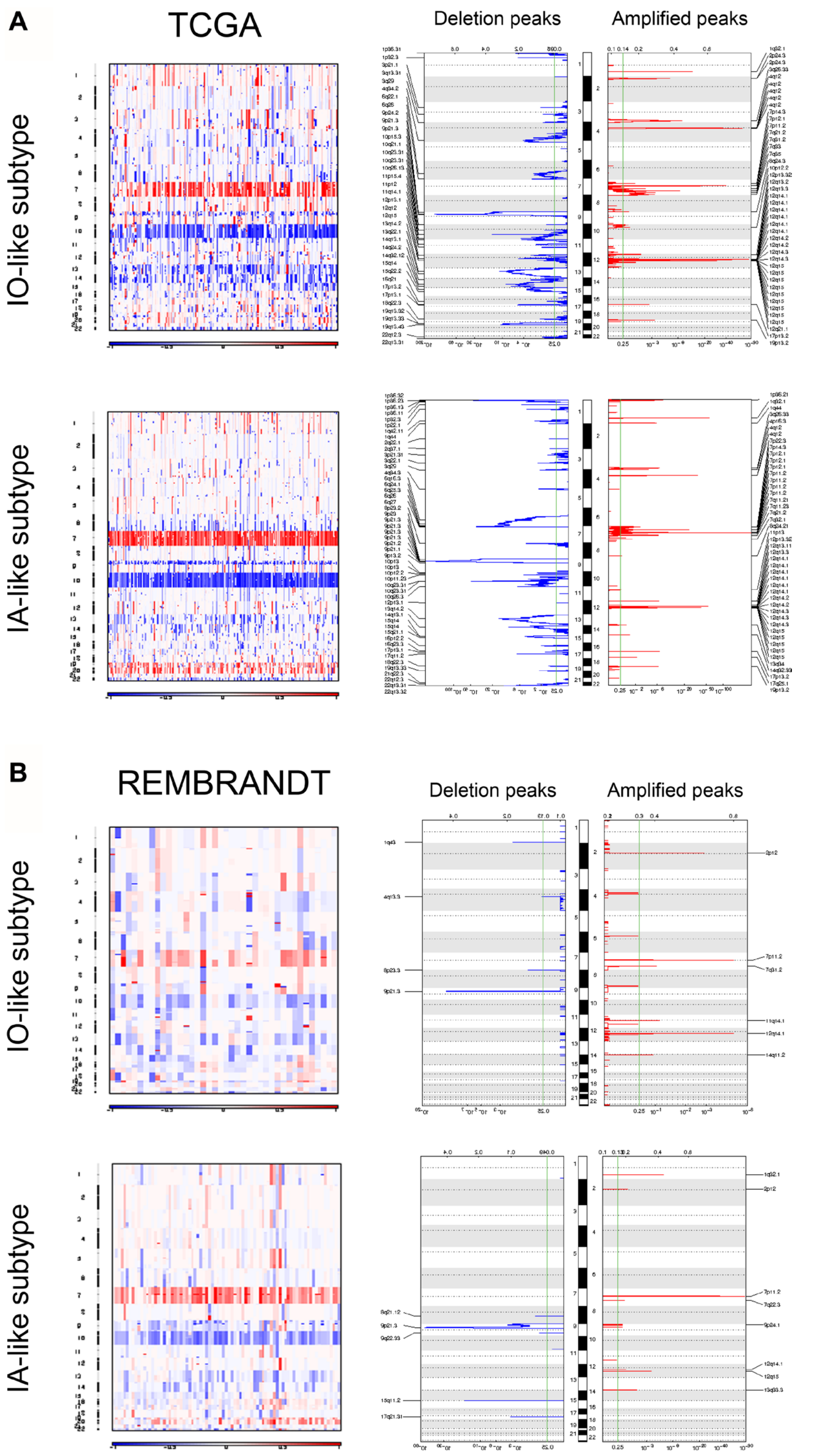

Supplementary Figure 5: Genomic copy number profiles of the gene network subtypes in (**A**) TCGA and (**B**) REMBRANDT; heat maps of genomic somatic copy number variations (SCNVs; *left panel*); plot for regional SCNVs (*right panel*).

Supplementary Table 1: Patient characteristics of the included datasets

Variables	Rennes–GSE22891	Rennes cohort	TCGA[a]	REMBRANDT	GSE50923	GSE16011	GSE36278	GSE60274
Sample size	50	56	570	177	52	144	57	79
Age (yrs)								
Median (range)	58 (26–80)	59 (36–75)	59 (18–89)	NA	57 (22–82)	55 (23–81)	45 (18–57)	51 (26–70)
KPS								
Median (range)	80 (40–100)	90 (50–100)	80 (10–100)	80 (20–100)	80 (50–90)	80 (30–100)	0	0
Unknown	4	22	133	98	0	2	57	79
Gender								
Male/female	25/24	40/16	367/203	79/48	30/22	97/47	34/23	59/20
Unknown	1	0	0	50	0	0	0	0
Extent of surgery								
Surgery (total/partial)	45 (36/9)	54 (41/13)	0	70 (33/37)	50 (30/20)	118 (44/74)	0	0
Biopsy	1	1	0	11	2	25	0	0
Unknown	4	1	570	96	0	1	57	79
Treatment regimens								
RT/TMZ	50	56	345	56	52	7	0	49
RT alone	0	0	117	8	0	106	0	30
Other or unknown	0	0	108	113	0	31	57	0

Yrs = years; RT = radiotherapy; TMZ = temozolomide; KPS = Karnofsky performance score; NA = not available.
[a]TCGA included 33 samples from secondary, recurrent or previously treated cases.

Supplementary Table 2: Results of Cox regression analysis within all available patients

Variables	Univariate Cox model			Multivariate Cox model		
	HR	95%CI	*P* value	HR	95%CI	*P* value
**All available cohorts[a, b] (*n* = 644)**						
Patient age	1.031	1.022–1.039	**< 0.001**	1.021	1.010–1.033	**< 0.001**
Three-CpGs signature	0.490	0.389–0.618	**< 0.001**	0.660	0.475–0.917	**0.013**
MGMT methylation status	1.797	1.479–2.182	**< 0.001**	1.752	1.355–2.266	**< 0.001**
G-CIMP status	3. 688	2.303–5.905	**< 0.001**	1.564	0.757–3.232	0.227
TCGA gene expression subtypes[c]	1.121	1.026–1.225	**0.011**			
Gender	0.784	0.639–0.963	**0.020**	0.713	0.554–0.918	**0.009**
Treatment[d]	0.420	0.318–0.553	**< 0.001**	2.416	1.735–3.364	**< 0.001**
All available cohorts[a, b] (*n* = 644)						
Patient age	1.031	1.022–1.039	**< 0.001**	1.021	1.009–1.034	**0.001**
Three-CpGs signature	0.490	0.389–0.618	**< 0.001**	0.772	0.504–1.036	0.077
MGMT methylation status	1.797	1.479–2.182	**< 0.001**	1.630	1.228–2.162	**0.001**
IDH1 mutations	3. 119	1.781–5.463	**< 0.001**	1.770	0.879–3.565	0.110
TCGA gene expression subtypes[b]	1.121	1.026–1.225	**0.011**	1.048	0.930–1.180	0.443
Gender	0.784	0.639–0.963	**0.020**	0.683	0.516–0.905	**0.008**
Treatment[e]	0.420	0.318–0.553	**< 0.001**	2.508	1.740–3.614	**< 0.001**

HR = hazard ratio; CI = confidence interval; KPS = Karnofsky performance score; G-CIMP = gliomaCpG island methylator phenotype;
[a]All available cohorts included GSE22891 (*n* = 50), Rennes cohort (*n* = 56), TCGA (*n* = 365; recurrent or secondary or previously treated cases were excluded), GSE50923 (*n* = 52), GSE36278 (*n* = 57), and GSE60274 (*n* = 64).
[b]Univariate and multivariate Cox models using each variable were also adjusted by different cohorts.
[c]TCGA gene expression subtypes includes mesenchymal, classical, proneural, and neural subtypes.
[d]Treatment included combination of RT/TMZ and RT alone.
In bold type were reported statistically significant results.

Supplementary Table 3: GSEA tables for the top enriched gene sets within low-risk or high-risk groups from TCGA. See Supplementary_Table_3

Supplementary Table 4: Characteristics of the EGFR/VEGFA/ANXA1-centered gene network classifiers. See Supplementary_Table_4

Supplementary Table 5: Gene set enrichment analysis (GSEA) on the up-regulated and down-regulated gene sets with respect to different risk tumors in each dataset

Dataset	Phenotype	Gene set	Size	NES	Nominal *p*-value	FDR *q*-value
TCGA[a] (*n* = 386)	Low-risk vs. high-risk	Up-regulated set	55	–2.24	< 0.001	< 0.001
		Down-regulated set	33	2.13	< 0.001	0.001
GSE60274 (*n* = 55)	Low-risk vs. high-risk	Up-regulated set	55	–1.59	< 0.001	0.020
		Down-regulated set	33	1.56	0.039	0.044
GSE22891 (*n* = 35)	Low-risk vs. high-risk	Up-regulated set	55	–1.41	0.112	0.220
		Down-regulated set	33	1.57	0.010	0.300
Rennes (*n* = 54)	Low-risk vs. high-risk	Up-regulated set	55	–2.10	0.002	0.002
		Down-regulated set	33	1.99	0.004	0.009

NES = Normalized Enrichment Score.

[a]TCGA samples with paired DNA methylation and gene expression data were included for analysis.

Supplementary Table 6: Gene set enrichment analysis (GSEA) of the signatures characteristic of different neruoglial lineages within different gene network subtypes in each dataset

Dataset	Phenotype	Classifier	Size	NES	Nominal *p*-value	FDR *q*-value
TCGA	IO-like vs. IA-like	Neurons	229	1.83	0.025	0.024
(*N* = 561)		Oligodendrocytes	216	1.65	0.069	0.070
		**Immature Oligodendrocytes**[a]	211	2.04	0.002	0.006
		Astrocytes	220	–1.75	0.012	0.025
		**Immature Astrocytes**[a]	222	–1.92	0.000	0.010
		Neural stem cells	49	–1.57	0.067	0.067
REMBRANDT	IO-like vs. IA-like	Neurons	234	1.62	0.000	0.031
(*N* = 177)		Oligodendrocytes	228	1.56	0.036	0.035
		**Immature Oligodendrocytes**[a]	218	1.75	0.000	0.010
		Astrocytes	225	–0.91	0.568	0.544
		**Immature Astrocytes**[a]	221	–1.33	0.111	0.275
		Neural stem cells	50	–1.14	0.339	0.360
GSE16011	IO-like vs. IA-like	Neurons	220	1.44	0.071	0.077
(*N* = 144)		Oligodendrocytes	218	1.45	0.09	0.18
		Immature Oligodendrocytes[a]	210	1.65	0.000	0.012
		Astrocytes	222	–1.20	0.219	0.250
		**Immature Astrocytes**[a]	222	–1.40	0.049	0.107
		Neural stem cells	48	–1.35	0.138	0.121
GSE7696	IO-like vs. IA-like	Neurons	234	1.46	0.058	0.153
(*N* = 70)		Oligodendrocytes	228	1.54	0.022	0.025
		**Immature Oligodendrocytes**[a]	218	1.69	0.000	0.003
		Astrocytes	227	–1.32	0.099	0.202
		**Immature Astrocytes**[a]	226	–1.52	0.006	0.032
		Neural stem cells	50	–1.19	0.269	0.256
GSE22891	IO-like vs. IA-like	Neurons	233	1.57	0.047	0.054
(*N* = 35)		Oligodendrocytes	225	1.65	0.023	0.061
		**Immature Oligodendrocytes**[a]	218	1.78	0.002	0.023
		Astrocytes	225	–0.86	0.627	0.605
		**Immature Astrocytes**[a]	225	–1.13	0.290	0.479
		Neural stem cells	50	–1.10	0.391	0.398
Rennes cohort	IO-like vs. IA-like	Neurons	234	1.60	0.071	0.060
(*N* = 54)		Oligodendrocytes	229	1.55	0.078	0.069
		**Immature Oligodendrocytes**[a]	224	1.99	0.011	0.007
		Astrocytes	228	–1.43	0.095	0.171
		**Immature Astrocytes**[a]	228	–1.54	0.079	0.206
		Neural stem cells	50	–1.01	0.333	0.320

NES = Normalized Enrichment Score; IO-like = immature oligodendrocyte-like; IA-like = immature asctrocyte-like;
[a] Among GSE9566, immature oliogodendrocytes were defined as non-myelinated oligodendrocytes and oligodendrocyte progenitor cells, and immature astrocytes were those harvested at postnatal day 1 to 8 days

Supplementary Table 7: GSEA tables for top enriched gene sets within the IA-like or IO-like clusters from each dataset. See Supplementary_Table_7

Supplementary Table 8: List of the significantly mutated genes within the IA-like and IO-like tumors from TCGA. See Supplementary_Table_8

Supplementary Table 9: List of the differentially expressed microRNAs between the subtypes from TCGA. See Supplementary_Table_9

Supplementary Table 10: List of the differentially expressed proteins between the subtypes from TCGA. See Supplementary_Table_10

Supplementary Table 11: Results of Cox regression analyses for the gene network clusters

Variables	Univariate Cox model			Multivariate Cox model		
	HR	95%CI	*P* value	HR	95%CI	*P* value
**TCGA[a] (*n* = 519)**						
Patient age	1.033	1.024–1.041	**< 0.001**	1.022	1.008–1.036	**< 0.001**
The gene network subtypes	0.746	0.603–0.923	**< 0.001**	0.602	0.433–0.836	**0.002**
MGMT methylation status	1.351	1.043–1.751	**0.007**	1.589	1.172–2.156	**0.003**
G-CIMP status	2.849	1.769–4.589	**< 0.001**	1.611	0.748–3.466	**0.223**
Gender	0.968	0.788–1.189	**0.755**			
Treatment b	0.359	0.280–0.462	**< 0.001**	0.442	0.317–0.617	**< 0.001**
**All available cohorts[a, c] (*n* = 1018)**						
Patient age	1.032	1.025–1.039	**< 0.001**	1.024	1.011–1.038	**< 0.001**
The gene network subtypes	0.686	0.591–0.796	**< 0.001**	0.593	0.431–0.814	**0.001**
MGMT methylation status	1.785	1.432–2.225	**< 0.001**	1.759	1.309–2.362	**< 0.001**
IDH1 mutations	2. 806	1.968–4.002	**< 0.001**	1.757	0.853–3.619	**0.126**
Gender	0.922	0.796–1.069	0.282			
Treatment[b]	0.417	0.333–0.521	**< 0.001**	0.431	0.299–0.622	**< 0.001**

HR = hazard ratio; C I = confidence interval; KPS = Karnofsky performance score; G-CIMP = gliomaCpG island methylator phenotype;
[a]TCGA samples obtained from recurrent or secondary or previously treated cases were excluded
[b]Treatment included combination of RT/TMZ and RT alone
[c]Univariate and multivariate Cox models using all available patients were all adjusted by different datasets
In bold type were reported statistically significant results.
