# Supplementary material for: Integrative analysis of novel hypomethylation and gene expression signatures in glioblastomas
Source: Oncotarget. 2017 Jul 11;8(52):89607–19. doi: 10.18632/oncotarget.19171 (PMC5685695; doi:10.18632/oncotarget.19171)
Supplement: Supplementary file 3 [file oncotarget-08-89607-s003.docx]

**Supplementary Table 4: Characteristics of the EGFR/VEGFA/ANXA1-centered gene network classifiers**

| Gene symbols | Gene set | Connectivity | Log2 fold change ^a^ | Chr. | Roles in GBMs | PMID ^b^ |
| --- | --- | --- | --- | --- | --- | --- |
| POSTN | upregulated | 1 | -2.94 | 13 | recruits M2 tumour-associated macrophages and promotes malignant growth | 25580734 |
| MEOX2 | upregulated | 1 | -1.84 | 7 | **Unclear functions** |  |
| FABP7 | upregulated | 1 | -1.84 | 6 | Glioma stem cell marker; promotes GBM cells proliferation and migration | 24274717;23284888 |
| LGALS3 | upregulated | 1 | -1.79 | 14 | promotes GBM cells survival in hypoxic and nutrient deprived microenvironments | 25369297 |
| PTX3 | upregulated | 1 | -1.43 | 3 | GBM-related inflammation | 23664694 |
| EGFR | upregulated | 25 | -1.40 | 7 | GBM core pathways members | 18772890 |
| TIMP1 | upregulated | 2 | -1.29 | X | promotes angiogenesis; unfavorable prognsoticator | 22177802;21163810 |
| ADM | upregulated | 3 | -1.29 | 11 | promotes GBM cells proliferation and angiogenesis | 19166930;21458987 |
| CHI3L1 | upregulated | 1 | -1.25 | 1 | chemo-resistance; radio-resistance; promotes angiogenesis and migration;mesenchymal markers | 25139333;24842123;24282289;21385870 |
| F3 | upregulated | 4 | -1.25 | 1 | promotes GBMs progression and angioegenesis | 19276385;12135696 |
| PLA2G5 | upregulated | 2 | -1.22 | 1 | **Unclear functions** |  |
| CDH4 | upregulated | 4 | -1.15 | 20 | bypass the cell-cell contact inhibitory signals | 24858041 |
| ITGA7 | upregulated | 5 | -1.15 | 12 | **Unclear functions**;somatic mutations in GBMs | 17551147 |
| PDGFA | upregulated | 1 | -1.15 | 7 | multiple GBM-promoting functions | 19832839 |
| VEGFA | upregulated | 16 | -1.15 | 6 | multiple GBM-promoting functions | 17643088;17882276 |
| SOCS2 | upregulated | 1 | -1.15 | 12 | chemo-resistance of GBM to Semustine | 21807073 |
| COL9A3 | upregulated | 4 | -1.12 | 20 | **Unclear functions** |  |
| EMP3 | upregulated | 1 | -1.12 | 19 | **Unclear functions**; overexpressed and lack of promoter hypermethylation in pGBMs | 17610521 |
| VAV3 | upregulated | 1 | -1.12 | 1 | enriched in glioma-initiating cells (GICs); invloves in GBM cells migration and invasion | 24832468;19008376 |
| RGS6 | upregulated | 8 | -1.09 | 14 | **Unclear functions** |  |
| P2RY1 | upregulated | 3 | -1.06 | 3 | promotes cell migration in C6 glioma cells | 21540076 |
| DPP4 | upregulated | 3 | -1.06 | 2 | contradictory oncogenic functions in GBMs | 17981714 |
| SPRY1 | upregulated | 3 | -1.03 | 4 | a negative regulator of GICs differentiation | 24155920 |
| VASN | upregulated | 1 | -1.00 | 16 | inhibits GBM cells apoptosis induced by hypoxia and inflammation (TNF-alpha) | 21658601 |
| SPRY2 | upregulated | 3 | -0.97 | 13 | potential driver mutations; promotes GBM cells growth and cellular resistance to RTKs inhibitors | 20406234;25934697 |
| PLAT | upregulated | 4 | -0.97 | 8 | promotes angiogenesis in C6 glioma cells; stem-like functions in GICs | 18535615;25627976 |
| GRB10 | upregulated | 2 | -0.97 | 7 | **Unclear functions;** amplified in GBMs | 19304958 |
| ANXA1 | upregulated | 12 | -0.97 | 9 | FoxM1 enhanced the proliferation, migration, and angiogenesis in Anxa1-dependent manner | 23991102 |
| B3GNT5 | upregulated | 2 | -0.94 | 3 | inverse correlation between promoter methylation and expression level in glioblastomas | 21156036 |
| VIM | upregulated | 3 | -0.92 | 10 | markers for epithelial-mesenchymal transition (EMT) | 18485877 |
| NOS2 | upregulated | 2 | -0.92 | 17 | multiple GBM-promoting functions | 25768009;25051975;21729780 |
| COL16A1 | upregulated | 4 | -0.89 | 1 | tumor-specific remodeling of the ECM; promotes cell invasion | 18804107;21471710 |
| IL8 | upregulated | 3 | -0.89 | 4 | multiple GBM-promoting functions especially pro-angiogenesis | 22139077;23538445;25200966 |
| RCAN1 | upregulated | 1 | -0.89 | 21 | attenuates NF-kappaB-mediated transcriptional activation | 17062574 |
| CAMK2B | upregulated | 1 | -0.86 | 7 | involved in brain tumor progression | 15750623 |
| PLA2G2A | upregulated | 3 | -0.86 | 1 | a link between GBMs and inflammation; activates EGFR signaling and promotes GBM cells proliferation | 20639215 |
| ANXA2 | upregulated | 4 | -0.86 | 15 | promotes angiogenesis and invasion in GBMs | 25697644; 23195957 |
| TGFB2 | upregulated | 4 | -0.84 | 1 | multiple GBM-promoting functions | 21619538 |
| IGFBP3 | upregulated | 4 | -0.84 | 7 | contradictory oncogenic functions in GBMs | 24878287;25059666 |
| CDH6 | upregulated | 4 | -0.84 | 5 | **Unclear functions** |  |
| LGALS1 | upregulated | 4 | -0.81 | 22 | GBM-related innate and adaptive immunity; TMZ-resistance;promotes angiogenesis and migration | 23929302;25744176;18431251 |
| TNC | upregulated | 4 | -0.81 | 9 | promotes angiogenesis and invasion in GBMs; marker for glioma stem cells | 21221826;25469866 |
| CXCL11 | upregulated | 9 | -0.81 | 4 | glioma cell tropism | 16434036 |
| LAMA2 | upregulated | 3 | -0.79 | 6 | promotesglioma stem cells growth | 23280793 |
| PDGFD | upregulated | 3 | -0.79 | 11 | chemo-resistance of GBM to Semustine;regulates survival and mitogenic pathways in GBM cells | 21807073;12097282 |
| NES | upregulated | 1 | -0.79 | 1 | markers for glioma stem cells | 15558011 |
| GALR1 | upregulated | 8 | -0.74 | 18 | responsible for most galanin-like binding immunoreactivity in GBMs | 12734662 |
| ITGA3 | upregulated | 6 | -0.74 | 17 | **Unclear functions** |  |
| ADAM12 | upregulated | 2 | -0.74 | 10 | **Unclear functions;** overexpressed and correlated with proliferative activity in GBMs | 15509542 |
| COL5A2 | upregulated | 7 | -0.71 | 2 | **Unclear functions;**GBM related genes by microarray analysis | 21114830 |
| ETV4 | upregulated | 3 | -0.71 | 17 | transcriptional factors; promotes GBM cells invasion | 17938207 |
| CDH2 | upregulated | 6 | -0.71 | 18 | markers for EMTs; promotes cell migration | 15527101 |
| CCL27 | upregulated | 8 | -0.69 | 9 | promotes GBM cells proliferation and invasion; Myeloid markers involved in anti-VEGF therapy response | 25149529;21632852 |
| TRIP12 | upregulated | 1 | -0.67 | 2 | **Unclear functions** |  |
| COL4A2 | upregulated | 6 | -0.67 | 13 | **Unclear functions**; amplified and overexpressed in GBMs | 17002787;12937144 |
| SV2C | downregulated | 1 | 0.58 | 5 | **Unclear functions** |  |
| ADRA2A | downregulated | 8 | 0.58 | 10 | **Unclear functions** |  |
| CALCRL | downregulated | 1 | 0.68 | 2 | **Unclear functions;**expressed in gliomas | 14747444 |
| SOX4 | downregulated | 1 | 0.76 | 6 | invloved in TGF-beta signaling and the stemness properties of glioma-initiating cells | 21987575;19896441 |
| CXCL12 | downregulated | 2 | 0.80 | 10 | promotes proliferation, invasion and angioegenesis in GBM cells and GSCs | 24561124;24442483;21618540;19738068 |
| CPEB3 | downregulated | 1 | 0.81 | 10 | **Unclear functions** |  |
| NPY | downregulated | 9 | 0.81 | 7 | **Unclear functions;**downregulated in C6 cell-induced glioma | 10357067 |
| BCL11B | downregulated | 1 | 0.82 | 14 | **Unclear functions** |  |
| CDKN2B | downregulated | 1 | 0.82 | 9 | hypermethylation and deletion in GBMs; tumor suppressor genes | 10574984;18772890 |
| ENPP2 | downregulated | 3 | 0.82 | 8 | **Unclear functions** |  |
| GPR17 | downregulated | 10 | 0.82 | 2 | promoting differentiation or quiescence of oligodendrocyte progenitor-like cells; antiproliferative effects | 22865458 |
| F5 | downregulated | 3 | 0.85 | 1 | **Unclear functions** |  |
| GRM1 | downregulated | 2 | 0.85 | 6 | **Unclear functions** |  |
| FGF12 | downregulated | 1 | 0.86 | 3 | **Unclear functions** |  |
| KCNJ3 | downregulated | 1 | 0.90 | 2 | **Unclear functions** |  |
| GALNT14 | downregulated | 3 | 0.93 | 2 | **Unclear functions** |  |
| DCX | downregulated | 2 | 0.93 | X | suppresses malignant phenotype; induces GSCs apoptosis;specifically expressed in neurons | 17178868; 21477071 |
| CDH13 | downregulated | 4 | 0.95 | 16 | PpIX biosynthesis in GBM cell lines; a negative regulator of neural cell growth | 24010971;10737605 |
| DNM3 | downregulated | 2 | 0.96 | 1 | **Unclear functions;**downregulated in GBMs by microarray analysis | 24440977 |
| EPHB1 | downregulated | 1 | 0.96 | 3 | suppresses invasion; promotes cell-cell adhesion | 24121831;23835497 |
| GNAI1 | downregulated | 4 | 1.04 | 7 | **Unclear functions** |  |
| FGF9 | downregulated | 3 | 1.05 | 13 | contradictory oncogenic functions in gliomas | 9696088;11801566 |
| SULF1 | downregulated | 1 | 1.10 | 8 | **Unclear functions** |  |
| LPAR4 | downregulated | 3 | 1.14 | X | **Unclear functions** |  |
| SH3GL3 | downregulated | 2 | 1.18 | 15 | glioma invasion-associated candidate genes | 21722156 |
| COL11A1 | downregulated | 6 | 1.24 | 1 | **Unclear functions;**upregulated in GBMs | 19351187 |
| FGF13 | downregulated | 1 | 1.32 | X | **Unclear functions** |  |
| SSTR1 | downregulated | 8 | 1.37 | 14 | reduces neovascularization and displays antiproliferative activity | 19706788;18566118 |
| VIPR2 | downregulated | 1 | 1.37 | 7 | inhibits GBM cells proliferation | 8923513 |
| SH3GL2 | downregulated | 4 | 1.37 | 9 | a tumor suppressor | 24736727;23029364 |
| CDKN2A | downregulated | 4 | 1.51 | 9 | hypermethylation and deletion in GBMs; tumor suppressor genes | 10574984;18772890 |
| GALNT13 | downregulated | 2 | 1.74 | 2 | **Unclear functions;** specifically expressed in neurons | 12407114 |
| CDH18 | downregulated | 4 | 1.74 | 5 | **Unclear functions** |  |

^a^ log2 fold change between high-risk and low-risk GBMs from TCGA

^b^ articles supported the roles of corresponding genes in GBMs
